# 4-D Computational Modeling of Cardiac Outflow Tract Hemodynamics over Looping Developmental Stages in Chicken Embryos

**DOI:** 10.3390/jcdd6010011

**Published:** 2019-02-27

**Authors:** Katherine Courchaine, MacKenzie J. Gray, Kaitlin Beel, Kent Thornburg, Sandra Rugonyi

**Affiliations:** 1 Department of Biomedical Engineering, Oregon Health and Science University, Portland, OR 97239, USA; courchai@ohsu.edu; 2 School of Public Health, Portland State University, Portland, OR 97035, USA; mackgray@pdx.edu; 3 Camas High School, Camas, WA 98607, USA; kaitlinbeel@gmail.com; 4 Knight Cardiovascular Institute, Oregon Health and Science University, Portland, OR 97239, USA; thornbur@ohsu.edu

**Keywords:** cardiovascular development, computational fluid dynamics, congenital heart defects, hemodynamics, outflow tract

## Abstract

Cardiogenesis is interdependent with blood flow within the embryonic system. Recently, a number of studies have begun to elucidate the effects of hemodynamic forces acting upon and within cells as the cardiovascular system begins to develop. Changes in flow are picked up by mechanosensors in endocardial cells exposed to wall shear stress (the tangential force exerted by blood flow) and by myocardial and mesenchymal cells exposed to cyclic strain (deformation). Mechanosensors stimulate a variety of mechanotransduction pathways which elicit functional cellular responses in order to coordinate the structural development of the heart and cardiovascular system. The looping stages of heart development are critical to normal cardiac morphogenesis and have previously been shown to be extremely sensitive to experimental perturbations in flow, with transient exposure to altered flow dynamics causing severe late stage cardiac defects in animal models. This paper seeks to expand on past research and to begin establishing a detailed baseline for normal hemodynamic conditions in the chick outflow tract during these critical looping stages. Specifically, we will use 4-D (3-D over time) optical coherence tomography to create in vivo geometries for computational fluid dynamics simulations of the cardiac cycle, enabling us to study in great detail 4-D velocity patterns and heterogeneous wall shear stress distributions on the outflow tract endocardium. This information will be useful in determining the normal variation of hemodynamic patterns as well as in mapping hemodynamics to developmental processes such as morphological changes and signaling events during and after the looping stages examined here.

## 1. Introduction

Presently, between 0.6 and 1.1% of babies born in the US have some form of congenital heart defect (CHD), 10–26% of which are classified as critical [1,2,3]. According to reports by the Centers for Disease Control and Prevention, CHDs are responsible for 4.2% of neonatal deaths and for treatment costs approaching $2 billion annually [4,5]. However, only about 15% of CHD cases can be directly attributed to chromosomal abnormalities [6], which points to other potential teratogens such as environmental toxins and maternal health conditions. These teratogens dysregulate normal cardiac development in a host of complicated ways, and the alteration of normal blood flow is emerging as one of the critical mechanisms by which congenital abnormalities originate. 

Blood flow, or hemodynamics, is increasingly recognized as a highly influential factor in normal embryonic development, particularly cardiovascular development. Blood passing over endothelial cells provides them with a pattern of mechanical stimuli in the form of pressure (normal force) and shear stress (tangential force). These stimuli also affect other cardiac cells, including myocardial and mesenchymal cells. Mechanosensors within cardiac cells respond to hemodynamic changes, both normal and aberrant, by activating mechanotransduction and downstream signaling pathways that modulate structural development via enabling processes such as cellular migration and proliferation [7]. Disrupted hemodynamic forces have been shown to initiate a host of cardiovascular developmental problems in and across animal models. Studies in mice have shown that yolk sac remodeling is directly affected by hemodynamics [8]. In chick embryos, mRNA expression in genes vital to valve development is altered; cushion formation, cardiac looping, and ventricular function are impaired; endothelial to mesenchymal transition is disrupted; and a variety of structural abnormalities resembling human CHDs occur under abnormal flow conditions [9,10,11,12,13]. In zebrafish, altered flow produces abnormal chamber formation, diminished looping, and impaired valve formation [14]. In fetal lambs, hemodynamic perturbation causes ventricular hyper- and hypoplasia and aortic stenosis [15]. The preceding list is inexhaustive but points to the fact that effects occur across models and indicates that mechanotransduction mechanisms are highly conserved among vertebrate species.

In the early embryonic stages, the heart begins as a linear tube and starts pumping blood soon after its formation. The tubular heart subsequently elongates, loops to form an s-shape heart tube, and eventually septates into a four-chambered heart. The looping stages (approx. 22–56 days in humans [7]) are both highly critical to normal development and exquisitely sensitive to flow conditions. Processes that occur during this window of development and that have been shown to depend on hemodynamic conditions include looping [14], ventricular trabeculation [16], and endothelial to mesenchymal transition [9,17]. The migration and proliferation of secondary heart field cells and neural crest cells also occur during this window [18,19]. The heart outflow tract (OFT), which is the most distal portion of the tubular heart, is a critical structure during looping stages, as it is involved in the development of the semilunar valves, interventricular septum, aorta, and pulmonary trunks [20]. Further, the majority of cardiac malformations in humans originate from the OFT. As such, the OFT has often been a structure of interest in studies that measure blood flow dynamics and/or their relation to experimental interventions, as well as molecular and structural changes under normal and experimental flow conditions [9,13,17,20,21,22,23,24,25].

Computational fluid dynamics (CFD) is emerging as an invaluable tool for quantifying flow parameters such as velocity and wall shear stress (WSS) in developing embryonic cardiovascular systems. CFD uses theoretical mathematical models, sometimes in tandem with experimental data, to provide detailed, multidimensional information about flow metrics that are difficult or impossible to assess completely or at all using direct measurement techniques. In subject-specific CFD models, the geometry of a cardiovascular structure of interest is extracted (segmented) from subject images, which may be collected, for instance, using confocal microscopy, micro-computed tomography, light sheet microscopy, or optical coherence tomography (OCT) [7]. The resulting geometry is then typically input into a computational software program, and boundary conditions are applied to specify flow characteristics. When applied to CFD models of the embryonic heart, this procedure presents two main challenges. First, the embryonic heart is very small and moves at a very rapid pace, making accurate spatiotemporal imaging resolution difficult to achieve. Geometries are therefore often taken from fixed tissue [9] or castings [23], which have the potential to have lost their true physiological shape in addition to lacking dynamic motion within an active cardiac cycle. The second challenge is that boundary conditions are not always possible to measure directly in the embryo being imaged, or at all, due to access and/or the embryos’ small size and high sensitivity to invasive procedures. Boundary conditions are therefore often estimated from previous studies or measured in other (matched) samples [26,27], which neglects individual factors affecting flow. Ideally, we would like to extract accurate 4-D (3-D through time) geometries and to adjust the boundary conditions so that they reproduce the hemodynamic physiology of the individual heart modeled.

This study overcomes the mentioned challenges in modeling developmental cardiac dynamics. We present a temporally detailed, embryo-specific CFD analysis of the normal chick OFT during a subset of the looping stages to gain insights into baseline blood flow conditions during cardiac looping. In chick embryos, stages are described using the Hamburger–Hamilton (HH) staging [28], with looping stages encompassing HH10 to HH24. We constructed CFD models of the OFT from HH14 to HH18, following the inverse modeling method outlined in Reference [29]: 4-D individual heart geometries are extracted from synchronized structural OCT images and model boundary conditions are iteratively adjusted to reproduce embryo-specific velocities measured using Doppler OCT. We also describe improvements made to our post-acquisition structural synchronization procedure since its initial publication in Reference [30]. During stages HH14 to HH18 (2–3 days of incubation), hematocrit is high enough that OCT, which relies on light scattering particles, can accurately measure blood velocity and the embryo is still small enough that the depth penetration of the system can reach the lower boundary of the OFT. Doppler OCT together with structural OCT allows us to simultaneously acquire vertical velocity data and structural images at high enough spatiotemporal resolution to accurately capture the motion of cardiac structures and the blood flow within them. CFD allows us to resolve the remaining two velocity components (Doppler OCT measures only the “vertical” velocity component) to quantify overall flow patterns, as well as create detailed 4-D maps of the wall shear stress distribution on the OFT endocardium.

## 2. Materials and Methods 

### 2.1. Imaging

Fertilized white Leghorn eggs were incubated blunt-end up until they reached the desired stages (HH14–18). Prior to imaging, a window was cut into the top of the eggshell and the vitelline membrane was peeled away, exposing the embryo. In order to preserve the humidity and visibility, a 0.13–0.16 mm glass coverslip (Karter Scientific, Lake Charles, LA, USA) treated with RainX antifog (Global Brands, Houston, TX, USA) was affixed over the window using low-temperature hot glue.

**Figure 1:**
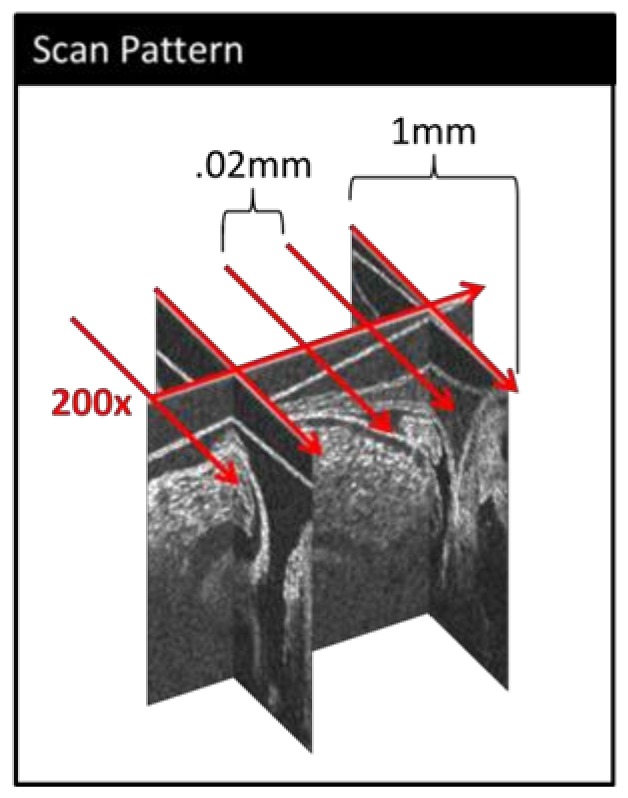
A simplified schematic of our custom 4-D optical coherence tomography (OCT) scan pattern. 200 b-modes (frames) are collected along the approximate centerline of the outflow tract (OFT) (longitudinal scan). Then, a series of 200-frame b-mode sequences are collected perpendicular to the initial scan (cross-sectional scans) at evenly spaced distances along the OFT.

Embryos were imaged with a custom-built OCT system (TELESTO-III-SP1, ThorLabs Inc., Newton, NJ, USA), using an in-house scan pattern. This scan pattern consists of a collection of b-mode (2-D image) sequences of 200 frames each, taken at 140 frames per second. This image acquisition rate is sufficient to acquire at least 50 frames per cardiac cycle during the stages examined here. The first b-mode sequence spans the length of the OFT (longitudinal scan), and then the scan line is rotated precisely 90 degrees and centered on the origin of the initial b-mode; subsequent b-mode sequences (cross-sectional scans) are collected perpendicular to the first dataset, moving along the length of the OFT in 20 µm increments (which separate each b-mode sequence), from the OFT inlet towards the outlet (Figure 1). Because temperature is very important for accurate physiological measurements in vivo, during imaging the eggs were placed in an ad hoc miniature incubator comprised of a ceramic cup and OMEGA thermocouple (OMEGA Engineering Inc., Norwalk, CT, USA).

### 2.2. Image Post-Processing

**Figure 2:**
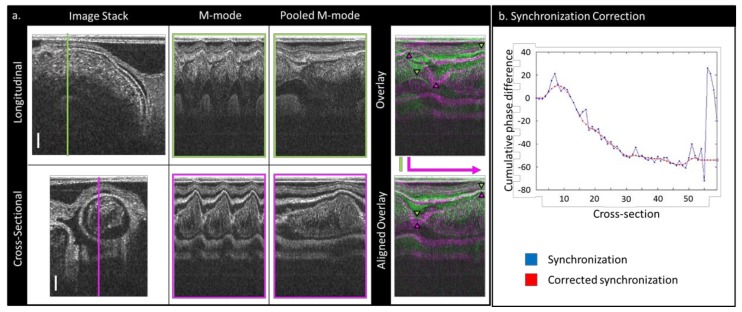
An illustration of the synchronization method employed to reconstruct 4-D OFT images from non-gated OCT datasets: (**a**) a schematic showing how m-modes are used to pool cardiac cycle information and to align cross-sectional datasets with the longitudinal dataset. Scale bars: 150 µm. (**b**) A plot showing an example of how the initial synchronization can be corrected and smoothed by the user: The phase scale is in units of pooled frames (1/200th of a cardiac cycle).

Acquired OCT images were post-processed using in-house MATLAB programs (The MathWorks, Inc., Natick, MA, USA) to achieve 4-D (3-D + time) embryo-specific image reconstructions. First, the OCT files were converted into structural image files and Doppler data files. Because the scan pattern did not use any gating techniques, both the images and the Doppler data required synchronization in order to reconstruct 3-D volume images over the cardiac cycle. This was accomplished using algorithms described in Reference [30], which have since been further developed to include an improved phase synchronization procedure based on longitudinal images. Due to the feature in our new OCT system that allows us to rotate our imaging plane at a specific pivot line, we were able to synchronize the cross-sectional sequences directly to the longitudinal sequence using m-modes generated at precisely correspondent positions. An m-mode is a 2-D depiction of motion in time, with image intensity along a line in a b-mode image (usually the depth) on the vertical axis and time on the horizontal axis. “Longitudinal” m-modes were generated from the longitudinal b-mode sequence by extracting a vertical column of pixels (spanning the depth of the OFT) at evenly distributed locations along the length of the OFT, correspondent to the locations of the cross-sectional sequences. Then, “cross-sectional” m-modes were generated by extracting the center vertical column of pixels in each image sequence, reciprocally correspondent to the location of the longitudinal scan m-mode. The cardiac period was determined from each m-mode using the string-length method explained in detail in Reference [30], and m-mode images were pooled (combined) into one normalized cardiac cycle. The phase shift between each cross-sectional (pooled) sequence and the corresponding longitudinal (pooled) sequence was determined such that it maximized the similarity between the two m-mode images (Figure 2a) [30]. Using this phase shift, each cross-sectional b-mode sequence and Doppler dataset was individually re-sequenced so that it would be synchronized to the corresponding longitudinal m-mode.

Following this initial synchronization, in order to reduce processing noise in the synchronized images, a separate set of m-modes was created from the pooled, re-sequenced cross-sectional datasets by extracting a horizontal line of pixels from across the center of the OFT as marked by the user. These m-modes were then compared to their adjacent neighbors, and their phase differences were established as before (by maximizing the similarity between the m-modes). The cumulative difference in phase along the OFT was then plotted so that the user could see and correct any discrepancies (Figure 2b), and a smoothed cumulative phase difference interpolation was obtained. The b-mode images and Doppler datasets were then re-sequenced to correspond to the smoothed cumulative difference in phase shift, interpolated to 100 frames, and saved. At this point, the collection of synchronized cross-sectional b-modes corresponded to the 3-D OFT geometry at 100 time points over the cardiac cycle, and the Doppler data files contained the relevant flow data. 

To verify the accuracy of the synchronization and to validate our procedure, vertical velocity data curves were extracted from the Doppler data files of both the original longitudinal dataset and the smoothed synchronized cross-sectional datasets at approximately four corresponding points along the OFT. The vertical velocity curves at the corresponding data points were compared between the datasets to determine the accuracy of the reconstruction.

### 2.3. Segmenting

**Figure 3:**
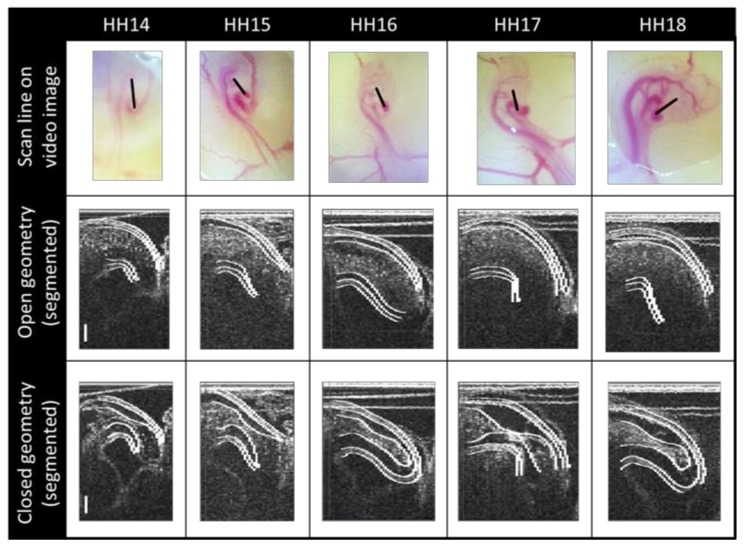
OCT image segmentation. **First row**: The longitudinal OCT scan location (black lines) superimposed on images of representative embryos at the stages considered. **Subsequent rows**: Representative structural OCT images reconstructed from synchronized cross-sectional datasets, with the results of our semiautomatic segmentation algorithm, shown here at a longitudinal section corresponding to the location of the black scan lines. Both open and closed OFT configurations are shown for each stage to illustrate the expansion and contraction of the OFT walls during the cardiac cycle which allows flow from the ventricle while blocking most backflow. Scale bars: 150 µm.

The resulting 4-D datasets were segmented using an in-house program delineated in Reference [31]. Briefly, our semiautomatic algorithm uses a two-dimensional deformable double-line model, a maximum likelihood estimator, and an active-contour model to extract first the myocardial layer and then the endocardial layer from 2-D images extracted from the reconstructed image volumes roughly perpendicular to the OFT centerline. The extracted endocardium geometries (Figure 3) represent the surface of the lumen at 100 time points during the cardiac cycle, from which 3-D geometrical models of the OFT over the cardiac cycle are constructed.

### 2.4. Computational Fluid Dynamics Simulations

Using the extracted dynamic geometries of the OFT lumen over the cardiac cycle, CFD simulations were performed with the commercial software ADINA (ADINA R&D, Inc., Watertown, MA, USA) on the high-performance computing Exacloud cluster at Oregon Health & Science University. The cardiac cycle of each embryo was normalized and divided into 100 time points (see Section 2.2. Image Post-Processing); cardiac OFT geometries at these time points were semiautomatically segmented (see Section 2.3. Segmenting), and the OFT segmentations were converted into hexahedral volume meshes with 8-node flow condition-based interpolation (FCBI) elements [29]. The volume meshes had an average of 61,195 elements; doubling the number of elements in two representative geometries did not yield significantly different results. Each timepoint was modeled using quasi steady-state conditions, with no-slip boundary conditions on the lumen walls. The assumption of the quasi-steady state is reasonable for most of the cardiac cycle, since the wall velocity is much smaller than the velocity of blood flow except for when the OFT is nearly closed or closed. In our simulations, Reynolds numbers were less than 5 (thus, flow inertia is negligible), and Womersley numbers were around 0.1 (thus, the phase lag between pressure drop and flow response is negligible), justifying our assumptions. Embryonic blood has a low enough hematocrit (approx. 15% compared to approx. 45% in adult humans [32,33]) at the stages examined here to be modeled as a Newtonian fluid using the Navier–Stokes equations, with a density of 1060 kg/m^3^ and a viscosity of 0.003 kg/ms [29]. Outlet and inlet geometries were artificially extended (with a smooth transition) in order to establish parabolic flow and to mitigate boundary effects (the extensions were not included in the post-simulation analyses). Boundary conditions were applied as follows: A zero-valued normal traction condition was applied to the outlet and a uniform, initially arbitrary normal traction was applied at the inlet in order to establish a pressure drop over the length of the OFT. Each time point underwent several iterations of simulation, wherein the computed velocity at a chosen location was compared to the measured velocity (extracted from the Doppler files) at the same location, and the inlet normal traction condition updated until the difference between the computed and measured velocities was <1%. Subsequent time points would use the final inlet condition of the previous point as a starting value. Details of this inverse method (i.e., matched velocity rather than inlet and outlet boundary conditions) can be found in Reference [29]. Finally, we compared the experimental and computed velocities at other points along the OFT (not used in the iterative procedure) in order to confirm the accuracy of the simulation over the entire geometry. This methodology ensured embryo-specific blood flow conditions were reproduced.

### 2.5. Geometrical Assessment and Stress Analysis

MATLAB and EnSight (CEI, Inc., Apex, NC, USA) were used to post-process and visualize the results of both the initial segmentation and the CFD simulation. The cross-sectional areas of the OFT (both lumen area and cardiac wall area, as defined by segmentation contours) change both in time (over the cardiac cycle) and space (along the OFT). This is due to the OFT shape and the motion of the heart tube. To better appreciate this cardiac wall behavior, we devised a 2-D visualization of this motion: area-motion plots [20,29]. In these plots, color-coded cross-sectional areas of the OFT are represented as a grid, with time on the horizontal axis and distance along the centerline on the vertical axis.

The volume of the lumen at each time point was estimated by multiplying the area of each segmented cross section by the distance between its centerpoint and its neighbor’s. This estimation allowed us to compute volumes even for closed geometries that could not be meshed and showed negligible difference (<2%) when compared with volumes calculated directly from reconstructed meshes. The centerline length reported in this paper was measured at a closed configuration of the lumen.

**Figure 4:**
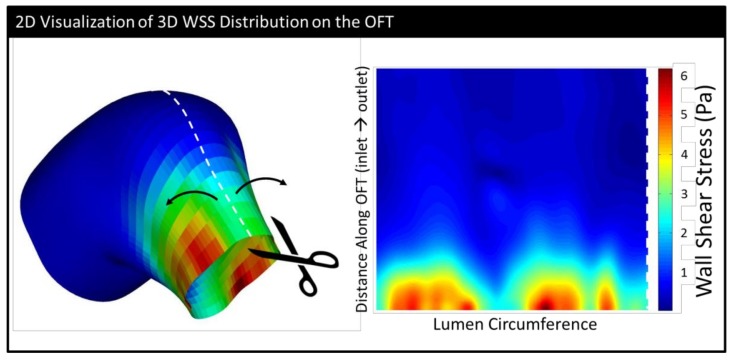
A schematic showing how the wall shear stress distribution on the endocardium is represented in 2-D. The surface of the endocardium is “cut” along the top and “unrolled” so that in the 2-D image, the inlet is at the bottom and the outlet is at the top. (The surface depicted is from an HH18 embryo at the point of maximum wall shear stress (WSS).)

We used EnSight to calculate the wall shear stress using the velocity gradients close to the wall from the CFD simulation results. Wall shear stress (|τ|) is calculated here as the magnitude of the traction vector (σ·n) projected onto the lumen surface:

(1) τ=σ·n − [(σ·n)·n]n

where ***σ*** is the flow stress tensor, ***n*** is a unit vector normal to the OFT wall, and τ is the projection of the traction vector on the OFT wall plane. Here, we also found it useful to make a 2-D representation of 3-D phenomena and analyzed the wall shear stress magnitude distributions |τ| on “unrolled” endocardial surfaces. These plots were designed to look as though someone sliced along the upper part of the heart tube and laid it flat (see Figure 4 and Video S1).

From the wall shear stress vector, τ, we also calculated the oscillatory shear index (OSI) for each embryo, using the formula

(2) $$\mathrm{OSI}\;=\;\frac{1}{2}\left(1-\frac{\left|\frac{1}{T}\int_{t}^{t+T}\tau \;dt\right|}{\frac{1}{T}\int_{t}^{t+T}\left|\tau \right|dt}\right),$$

where T is the simulated cardiac cycle length. OSI ranges from 0 to 0.5, with 0 corresponding to flow that is consistently unidirectional (no backflow), and 0.5 indicating oscillatory flow with equivalent forward and backward flow [34].

Volume flow rates, Q, were calculated using a built-in EnSight function and compared across embryos. From these, we calculated forward flow volume, V_F_ (the total volume of blood going from the ventricle to the arterial system during the cardiac cycle); backward flow volume, V_B_ (the total volume of blood that goes back to the ventricle over the cardiac cycle); and overall stroke volume, SV (SV = V_F_ − V_B_). We also calculated a cardiac efficiency index (η) as follows:

(3) $$\eta =1-\;\frac{V_{B}}{V_{F}}$$

### 2.6. Embryonic Sex Determination

The sex of each embryo was determined via the polymerase chain reaction (PCR) amplification of avian sex chromosomes, as the sex of early stage avian embryos is not visually observable. Post-scanning, leg-bud tissue was harvested from each embryo, and DNA was extracted using the “Quick DNA purification protocol” from the Jackson Labs website (jax.org). Birds have Z and W sex chromosomes; male birds are homogametic (ZZ) and females are heterogametic (ZW) [35]. We used the primer pair (2718F/2524R) and PCR protocol in Reference [36] to amplify the homologous regions of two highly conserved Chromo-Helicase-DNA binding (CHD) genes located on both the Z and W avian sex chromosomes. A male embryo (ZZ) was identified by a single bright band at 650 base pairs corresponding to the CHD gene on the Z chromosome (CHD-Z). A female embryo (ZW) was identified by a band at 650 base pairs (CHD-Z) and an additional band at 450 base pairs, corresponding to the W-chromosome (CHD-W).

## 3. Results

4-D OCT scanning, synchronization, segmentation, and embryo-specific simulations were performed for a total of 16 embryos spanning the cardiac looping stages: three HH14; one HH15; and four each of HH16, HH17, and HH18 embryos. Due to meshing constraints, we were not always able to simulate the more closed lumen configurations, when the cushions are fully coapted and in contact with each other; in all cases, however, >60% of the cardiac cycle was simulated. The data means were compared in Excel, using two-sample t-tests assuming either equal or unequal variance based on the results of an *f*-test, although we were unable to compare HH15 to the other groups using this method as we had only one embryo that fell into this stage. We had relatively equal sex representation within each staging group as well as for our whole cohort (Table 1). Although our data is too limited to draw sweeping conclusions, it did not show any obvious differences between male and female embryos (Figure S1).

### 3.1. Physiology and Geometry

**Figure 5:**
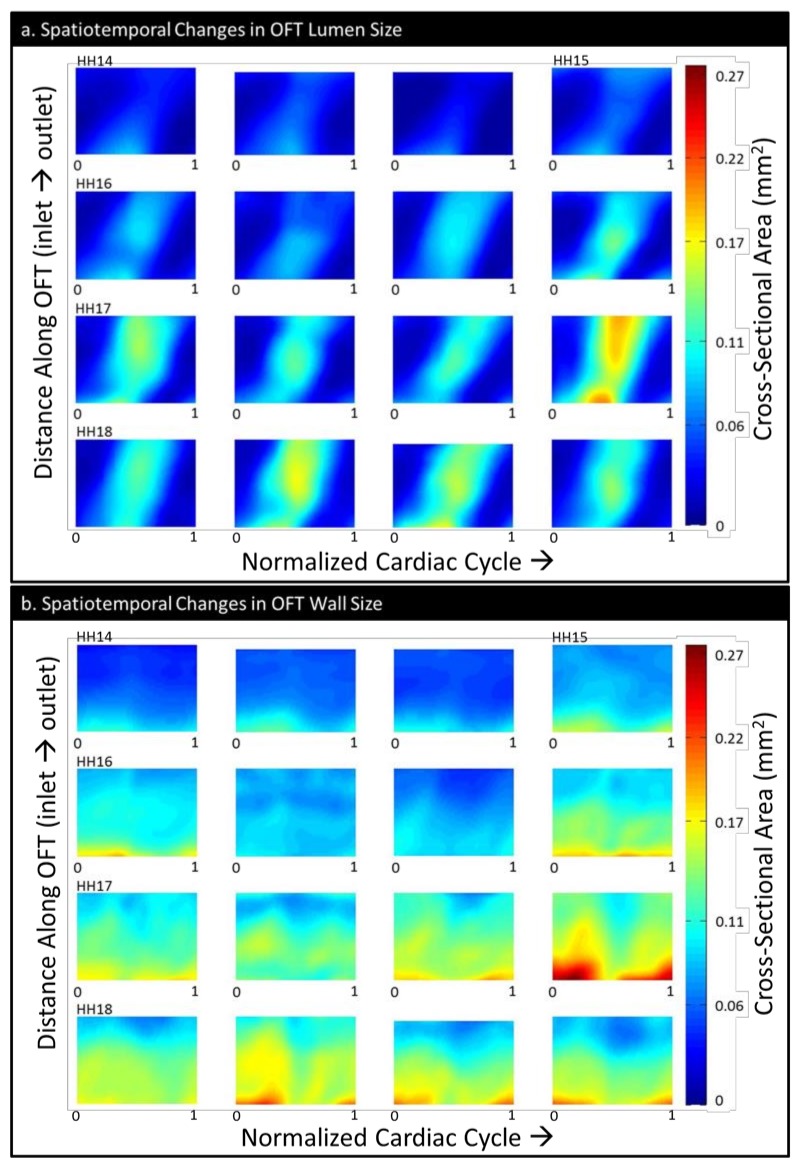
The OFT size depicted as the color-coded area of its cross sections over time (normalized cardiac cycle, horizontal axis) and space (normalized OFT length, inlet to outlet, vertical axis): (**a**) the spatiotemporal changes in the OFT lumen cross-sectional area and (**b**) the spatiotemporal changes in the OFT wall (cushions plus myocardium) cross-sectional area. The embryos have been ordered from youngest to oldest within each stage group and aligned in rows by stage.

The *cardiac cycle lengths* decreased with increasing stage (Table 1). The value ranges were consistent with those reported in Reference [20] and comparable to those from Reference [37]. Consistent with the growth of the OFT, the *maximum lumen volume* increased with stage, as did the *mean lumen volume* (Table 1). The *centerline length* appeared to increase until HH17, with a slight decrease in the centerline length for HH18, likely due to the decrease in visibility at the outlet as the embryo expands. The HH16, HH17, and HH18 groups did not significantly differ from each other in maximum volume or centerline length. However, HH14 had a significantly lower *maximum volume* compared with HH16, HH17, and HH18 (*p* = 0.010, *p* = 0.001, and *p* = 0.010, respectively), as well as a lower *centerline length* than HH16–18 (*p* = 0.013, *p* = 0.024, and *p* = 0.033) (Table 1). HH16 had a slightly lower *mean volume* than HH17 (*p* = 0.026), and HH14 also had a lower mean volume than HH16, HH17, and HH18 (*p* = 0.006, *p* < 0.001, and *p* = 0.007). Size as quantified by area-motion plots (Figure 5) also showed an increase in both lumen and cardiac wall area with stage. Motion of tissue in the longitudinal direction (along the heart OFT length) is apparent, and area-motion plots of the lumen, normalized to both OFT length and cardiac cycle length, visually indicate that the phase lag (time between points of maximum expansion) from inlet to outlet decreases only very slightly with stage (Figure 5). Generally, the lumen cross-sectional area increases with stage, as does the cardiac wall area. There was considerable variability within staging groups for all size measurements.

### 3.2. CFD

**Figure 6:**
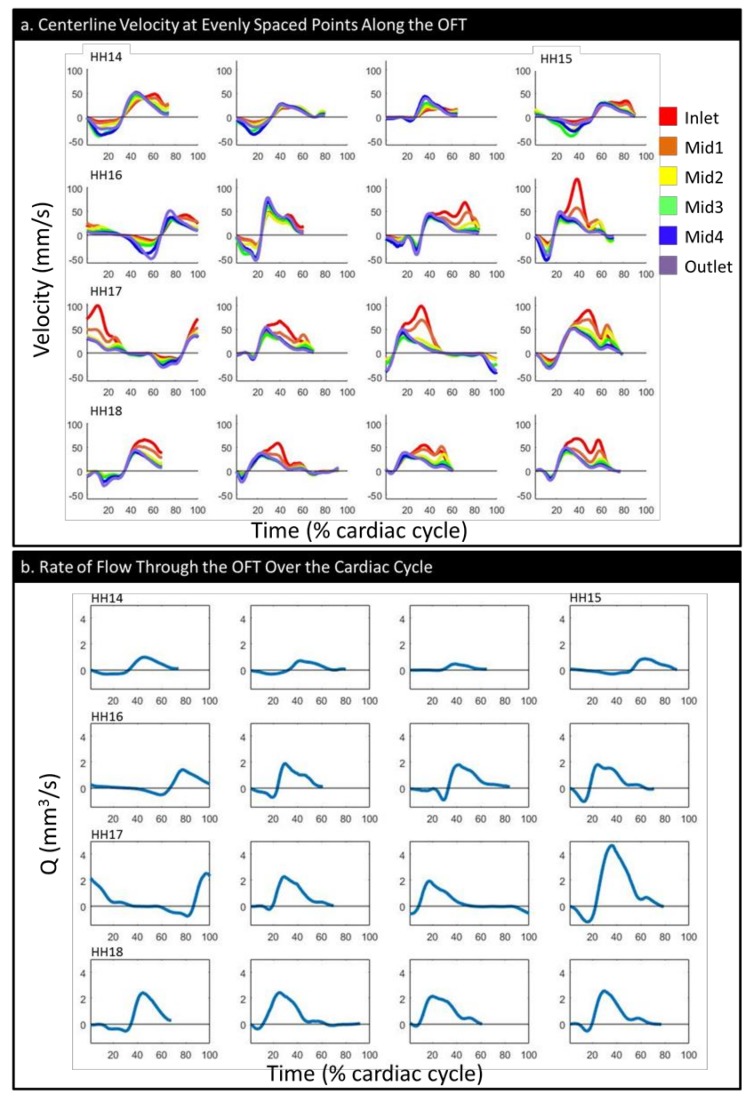
The flow parameters over the simulated portion of the normalized cardiac cycle: (**a**) the velocity magnitude at evenly spaced points along the centerline and (**b**) the flow rate (Q). The embryos have been ordered from youngest to oldest within each stage group and aligned in rows by stage.

The examination of velocities along the centerline showed that at earlier stages, velocity was higher at the outlet and transitioned during HH16 to being higher at the inlet for the majority of the cycle (Figure 6a), coincident with an increased OFT cushion size (see Figure 5b). The maximum *forward velocity* had an arc-like trend, with values increasing from HH14 to HH17 (*p* = 0.007) and decreasing between HH17 and HH18 (*p* = 0.015), although HH18 still had a higher forward velocity than HH14 (*p* = 0.044). The *forward flow volume*, V_F_, generally tended to increase with embryonic stage (Table 1 and Figure S1b); the differences were significant between HH14 and HH16–18 (*p* = 0.006, *p* = 0.049, and *p* < 0.001) and between HH16 and HH18 (*p* = 0.006).

All of our embryos showed at least some small period of backflow, but the portion of the cardiac cycle presenting backflow decreased as embryonic stage increased (Figure 6a,b). The peak magnitude of the *backflow velocity* had a wide range of values in each stage; however, it showed a clear decreasing trend following HH16. Changes were significant between HH16 and HH17 (*p*= 0.021) and between HH16 and HH18 (*p* = 0.003) (Table 1). However, the *backflow volume* (V_B_, the total volume of flow going back toward the ventricle over the cardiac cycle) did not have an obvious trend (Table 1, Figure S1a).

The *total stroke volume* (Figure S1c), *cardiac efficiency* (Figure S1d), and *maximum flow rate* (Figure 6b, Figure S1e) all tended to increase with embryonic stage (Table 1). The stroke volume had a significant difference between HH14 and HH16–18 (*p* = 0.005, *p* = 0.048, and *p* < 0.001) and between HH16 and HH18 (*p* = 0.005), as did maximum flow rate (*p* = 0.002, *p* = 0.045, *p* < 0.001, and *p* = 0.003). The cardiac efficiency showed a significant difference only between HH16 and HH18 (*p* = 0.017).

**Figure 7:**
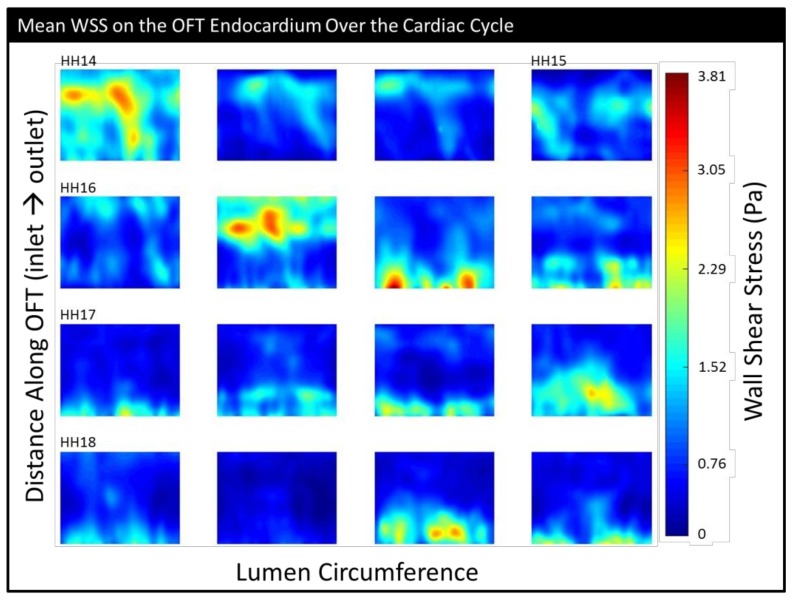
The mean (temporally averaged) wall shear stress over the simulated portion of the cardiac cycle shown on an “unrolled” endocardium surface (inlet at the bottom and outlet at top). The embryos have been ordered from youngest to oldest within each stage group and aligned in rows by stage.

*Maximum WSS* and *mean WSS* showed the highest variability at HH16, perhaps indicating a transitional period (Table 1, Figure S1f,g). At HH16, areas of intense WSS appeared to transition from being relatively evenly distributed along the OFT and/or near the outlet at the younger stages to being more heavily concentrated at the inlet at later stages; regions of high WSS also tended to be more compact at later stages (Figure 7). Embryos with the highest maximum WSS did not necessarily have the highest mean WSS. The mean WSS was significantly different between HH16 and HH18 (*p* = 0.036), and the maximum WSS was significantly different between HH14 and HH17 (*p* = 0.016).

Both the *mean (spatially averaged) OSI* and *maximum OSI* had no significant differences between stages. As stages progressed, however, more embryos featured low OSI indicative of mostly forward flow (<0.2; Figure S1h) and higher cardiac efficiency (>0.8; Figure S1d).

## 4. Discussion

The primary limitation of our study is the natural movement of the embryo during the scan period. Because the heart volume scanned is so small (approx. 1.2 × 1 × 1.2 mm) and the embryo itself is small and very sensitive, anything from temperature fluctuations to the angle of the surface of the yolk after the egg is placed under the scanner to the reverberations of its own heartbeat can cause the embryo to move. Although we made every effort to keep the egg level and at a stable temperature and the scan time as short as possible (~4 min) given the amount of data collected, many of the embryos we scanned (about 35%) exhibited enough motion to be immediately discarded or to completely derail the synchronization algorithm, at which point they were discarded. The scans that were successful and that are presented here very likely experienced some slight embryo movement as well, which has the potential to affect the resulting segmented geometry. However, these motions were negligible, as we were able to visually monitor the embryo movement throughout the scanning process using video images (approx. 0.014 mm/pixel) taken at the beginning of each b-mode sequence, and from those video images, no embryo motion was visually detected.

This study was designed to further our understanding of spatiotemporal wall shear stress and velocity distributions within the normal chick embryonic outflow tract during a subset of the looping stages of cardiac development (HH14–18). Morphological changes during cardiac development have been extensively studied, but the changes in blood flow dynamics and hemodynamic stimuli that take place have yet to be well-characterized. The objective of the study was to determine the hemodynamic baseline associated with cardiac growth during looping stages. While we acknowledge that an analysis of more embryos would have been desirable, our study is a stepping stone in characterizing hemodynamics and hemodynamic transitions during looping stages. We plan to expand our sample sizes and to build upon these initial results in order to establish a robust baseline for normal hemodynamic parameters within the chick heart outflow tract.

We used an established and validated inverse modeling technique [29] to generate a 4-D computational fluid dynamics model of the OFT over the cardiac cycle in a total of 16 embryos staged HH14 to HH18. These stages have been shown to be essential to normal cardiac development and are exquisitely sensitive to hemodynamic perturbation; several studies have implicated flow disturbances during this period with dramatically increased incidence of congenital heart defects [12,13,38]. Embryo-specific CFD studies such as this one can provide significantly more detailed and accurate velocity flow profiles and WSS mappings than would be estimable using OCT alone (or ultrasound, etc.), as CFD models resolve all three velocity components and take into consideration more precise geometrical information. The ability to generate fully three-dimensional WSS maps over the full cardiac cycle can provide critical insight into how spatial variations in shear forces are disrupted during experimental hemodynamic perturbations that cause malformations in structures that arise from the OFT, e.g., vitelline vein ligation or left atrial ligation [12]. It also provides a crucial reference for the extent of normal variation in WSS distribution. In particular, establishing such a detailed profile of the baseline hemodynamic environment in normal embryos would also be a significant step in creating a direct mapping of wall shear stress and other hemodynamic cues to signaling events, morphological changes, and other developmental processes that ultimately shape the heart and determine cardiac tissue function.

Our results were consistent with the (limited) existing literature. As previously mentioned, our cardiac cycle lengths (which change with stage and are also heavily influenced by temperature) were within the ranges reported in Reference [20] and comparable to those from Reference [37]. Velocities in the middle of the OFT at HH17 and HH18 were comparable to values reported in Reference [20]; our peak velocity at HH17 was comparable to that reported in Reference [9]. Although the computational model in Reference [20] had a maximum velocity magnitude that was about 65% of ours at HH18, the one from Reference [26] was 30% higher. Our peak velocities for HH16 were centered on the value reported in Reference [23] for that stage; their WSS values, however, were considerably lower. This may be, in part, due to their use of a fixed-tissue model at an open configuration (maximum WSS does not necessarily occur at the point of maximum expansion), and their model also appears to only consider the proximal region of the OFT. We report WSS values at HH18 that are comparable to those from References [9] and [26]. Our reported quantifications, therefore, are consistent with those from our group and others.

We observed minimal change in normalized phase lag over the stages studied (Figure 5a, slope of maximum area). That is, the wave-like motion of the OFT walls is preserved across stages. Since cardiac cycle length decreases with stage and the OFT length increases, this may be interpreted to mean that there are compensatory mechanisms in place such that the motion of the contraction/expansion wave within the OFT relative to the overall cardiac cycle and OFT length remains similar over developmental stages (HH14 to HH18).

Based on these results and results from previous studies that looked at flow parameters over the looping stages, HH16 seems to be a particularly critical period of transition. For example, Reference [20] showed the highest variance in cardiac cycle length at HH16, after which the rate of decrease in the cycle length appeared to level out; the time of flow (the portion of the cardiac cycle during which blood flow is observed) also began increasing after HH15. Their data also showed that the stroke volume and peak flow began increasing rapidly between HH15 and HH16 and continued to do so through HH18. The maximum velocity peaked at HH16 and then was stable. In Reference [37], the mean dorsal aortic blood flow was higher relative to the weight of the embryo and the vascular bed at HH16 than it was at HH14 or HH18, as was the ventricle weight relative to the embryo weight. Our data both confirms these prior results and adds important information about hemodynamics during this transition period. The centerline velocity measurements (Figure 6a), which we were able to calculate in their entirety (that is, fully in 3-D) using CFD, showed that during HH16, regions of higher velocity transitioned from occurring near the outlet to occurring at the inlet. WSS also followed this pattern, with a transition around HH16 from relatively disperse, outlet-skewed WSS distributions to clusters of high WSS values near the OFT inlet, as seen in our detailed endocardial surface maps (Figure 7). Following HH16, the embryos start featuring reduced backflow, while the stroke volume is increased, resulting in a transition to decreased OSI and increased cardiac efficiency (Figure S1). These transitions likely occur as a result of the increases in endocardial cushion volume (Figure 5b), which render the cushions more effective in restricting backflow. Hemodynamic transitions around the endocardial cushions, in turn, influence further endocardial cushion development at cellular and structural levels, including the progression of endocardial to mesenchymal transition, which starts around HH16 in the OFT [17] and later valve development [12]. As a whole, our data provide a unique lens to show how from HH14 to HH18, HH16 is a period of hemodynamic transition in the heart. These trends are of significant importance in planning future studies that involve flow perturbation in and around stage HH16, as they suggest critical changes in the function of the heart structure and its hemodynamic environment.

## Figures and Tables

**Table 1:** A summary of the heart outflow tract hemodynamic results. The values are presented as the mean plus or minus the standard deviation.

	HH14	HH15	HH16	HH17	HH18
	(*n* = 3)	(*n* = 1)	(*n* = 4)	(*n* = 4)	(*n* = 4)
# Male	2	0	3	2	1
Cardiac cycle (ms)	486 ± 54	507	408 ± 35	410 ± 30	400 ± 35
Maximum lumen volume (mm^3^)	03 ± 005	04	07 ± 02	095 ± 02	09 ± 02
Mean lumen volume (mm^3^)	02 ± 0.004	02	04 ± 01	05 ± 01	05 ± 01
Centerline length (mm)	54 ± 01	59	73 ± 07	78 ± 12	69 ± 0.08
Maximum WSS (Pa)	6.3 ± 7	6.3	11.0 ± 5.5	10.6 ± 2	7.9 ± 3.4
Mean WSS (Pa)	1.1 ± 5	97	1.1 ± 22	9 ± 0.17	75 ± 0.20
Mean oscillatory shear index (OSI)	25 ± 11	31	29 ± 03	21 ± 0.12	16 ± 0.13
Maximum OSI	45 ± 08	49	49 ± 01	38 ± 0.16	3 ± 0.22
Maximum backflow velocity (mm/s)	30 ± 19	40	50 ± 6	30 ± 12	21 ± 11
Maximum forward velocity (mm/s)	43 ± 12	35	82 ± 29	90 ± 15	62 ± 6
Backflow volume (mm^3^/beat)	02 ± 02	04	04 ± 002	04 ± 0.02	02 ± 0.02
Forward volume (mm^3^/beat)	07 ± 02	1	15 ± 02	25 ± 0.11	21 ± 0.02
Stroke volume (mm^3^/beat)	05 ± 01	06	12 ± 02	21 ± 0.1	19 ± 0.03
Cardiac efficiency	75 ± 19	64	76 ± 02	84 ± 0.1	92 ± 0.08
Maximum flow rate (mm^3^/s)	72 ± 6	87	1.7 ± 0.21	2.8 ± 1.24	2.4 ± 0.17

## Supplementary Materials

The following are available online at https://www.mdpi.com/2308-3425/6/1/11/s1, Video S1: The corresponding 3-D and 2-D videos of WSS over the cardiac cycle in an HH17 embryo, Figure S1: The hemodynamic parameters by stage and sex.
