# Supplementary material for: 4-D Computational Modeling of Cardiac Outflow Tract Hemodynamics over Looping Developmental Stages in Chicken Embryos
Source: J Cardiovasc Dev Dis. 2019 Feb 27;6(1):11. doi: 10.3390/jcdd6010011 (PMC6463052; doi:10.3390/jcdd6010011)
Supplement: Supplementary file 1 [file jcdd-06-00011-s001.zip › Supplementary figures and captions.docx]

Supplementary figures and captions:

***see attachment*
Video S1.** The corresponding 3-D and 2-D videos of WSS over the cardiac cycle in an HH17 embryo, with the cardiac cycle illustrated via the maximum velocity plot in the upper left.

**
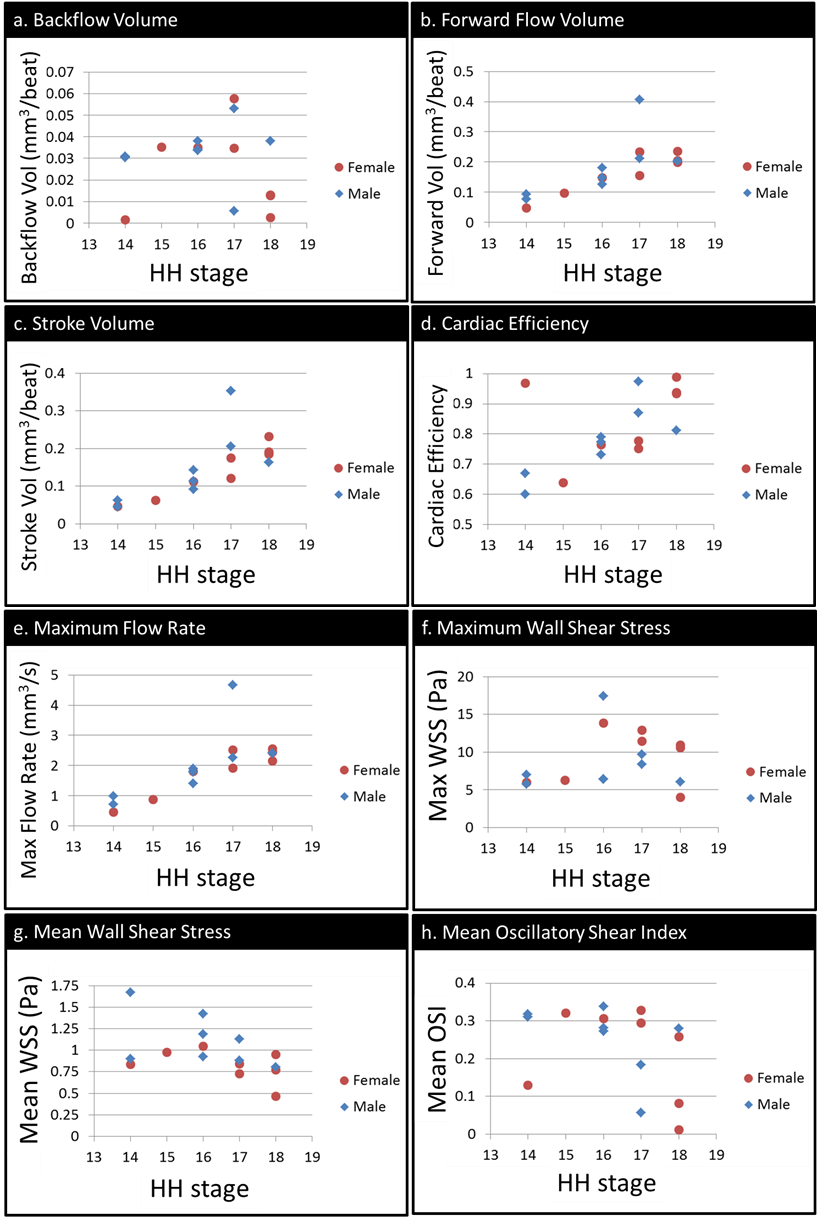

Figure S1.** The hemodynamic parameters by stage and sex: **a.** the backflow volume; **b.** the forward flow volume; **c.** the stroke volume; **d.** the cardiac efficiency; **e.** the maximum flow rate (Q); **f.** the maximum wall shear stress (WSS); **g**. the mean (spatiotemporally averaged) wall stress; and **h**. the mean oscillatory shear index (OSI).
